# Quality of COVID-19 research: An overview of large-scale assessments and a case study on excess mortality estimates

**DOI:** 10.1093/eurpub/ckac129.126

**Published:** 2022-10-25

**Authors:** L Belbasis

**Affiliations:** METRIC-B, QUEST Center, BIH, Charité – Universitätsmedizin Berlin, Berlin, Germany

## Abstract

COVID-19 is an ongoing public health emergency, which affected individual and population health, whereas it disrupted social and economic activities for more than 2 years. High-quality research evidence, which is published without delays in the peer-review and publication pipeline, is the most powerful tool for evidence-based decision-making by physicians, public health and health policy specialists, and politicians. However, from the early stages of the pandemic till now, there is a debate on the quality of COVID-19 research, whereas it has been observed that the decrease in the time from submission to publication was accompanied by a decrease in research quality. This presentation discusses the findings and main conclusions from two research projects. The first part focuses on a systematic review of published large-scale qualitative evaluations of COVID-19 research. By taking advantage of these large-scale assessments, we will provide a bird's eye view on the quality of COVID-19 research done so far. The second part focuses on a case study describing the methodological quality of studies calculating excess mortality estimates during COVID-19 pandemic. Excess mortality estimates depend on important choices about the pre-pandemic reference period, the pandemic period of interest, and the modelling of the comparison between these two periods. Such an assessment has not been done yet although there is an abundance of published approaches to estimate excess mortality during the COVID-19 pandemic. Overall, these two projects highlight the importance of meta-research in the time of pandemic by scrutinizing and assessing scientific evidence while deriving recommendations for improvement of future research. This presentation concludes with a summary of the implications of low-quality COVID-19 research in decision-making and some general considerations to improve research quality and integrity in the case of future pandemics.

